# An Atypical Presentation of Acoustic Neuroma With Facial Paresthesia: A Case Report

**DOI:** 10.7759/cureus.56745

**Published:** 2024-03-22

**Authors:** Shahul Irfan, Amogh D Kadam, Umarani Ravichandran

**Affiliations:** 1 Internal Medicine, Government Medical College and Hospital Cuddalore, Chidambaram, IND

**Keywords:** trigeminal nerve, brain tumor, vestibular schwannoma, facial paresthesia, acoustic neuroma

## Abstract

Acoustic neuromas are benign neoplasms of the brain composed of Schwann cells, arising most commonly from the nerve sheath of the vestibular division of the VIII cranial nerve. They usually manifest as unilateral hearing loss, tinnitus, and unsteadiness. Some patients may present atypically with symptoms like orofacial pain, hemifacial numbness, sudden onset hearing loss, or trigeminal neuralgia. Here we report an interesting case of acoustic neuroma in which the patient presented with unilateral facial numbness and tooth pain. Persistent atypical symptoms should always raise clinical suspicion of this pathology, necessitating the need for higher radiological investigations (CT or MRI) to aid in the early diagnosis and treatment.

## Introduction

Acoustic neuromas are frequently occurring non-malignant tumors within the brain, with an incidence ranging from 0.6 to 1.9 per 100,000 population, and account for 6% of all brain tumors [1]. They arise within the internal acoustic meatus but expand in the medial direction through the meatal orifice into a potential space formed between the cerebellum and pons (cerebellopontine angle), compressing the roots of various cranial nerves. This leads to the development of symptoms related to the compression of those cranial nerves [2].

Due to its insidious growth rate of 0.99-1.11 mm/year, the presentation of symptoms occurs at a later stage of the disease [3]. As the tumor arises commonly from the vestibulocochlear nerve, symptoms of compression of that nerve are the early features. However, when the tumor encroaches the superior part of the cerebellopontine angle, it causes compression and demyelination of the trigeminal or facial nerve leading to atypical symptoms like facial pain, numbness, oromandibular pain, or, in rare cases, facial paralysis [4]. Large neuromas can cause compression of the brain stem leading to pressure effects causing nausea, vomiting, or diplopia [5]. To date, there are very few case reports of acoustic neuroma presenting with atypical symptoms.

## Case presentation

**Figure 1:**
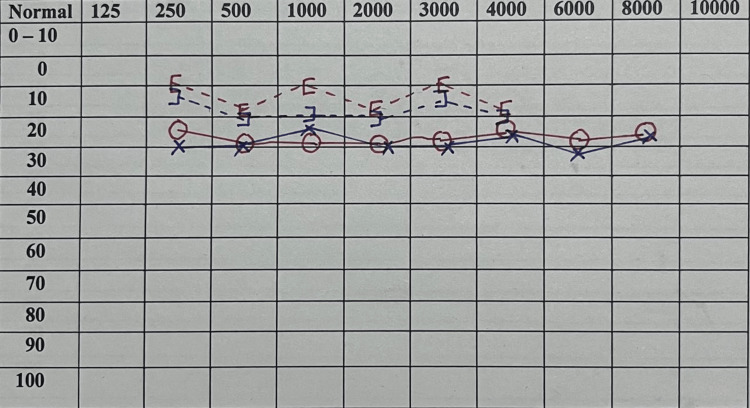
Pure-tone audiometry reveals no evidence of conductive or sensorineural hearing loss.

A 52-year-old female presented to the internal medicine outpatient department with a five-month history of numbness of the right cheek and right-sided tooth pain. The pain was gradual in onset, intermittent in nature, and aggravated by opening the mouth and chewing. The patient reported no prior personal or family history of similar complaints. Cranial nerve examination revealed a decrease in sensation of the skin supplied by the ophthalmic (V1 ) and maxillary (V2) divisions of the right trigeminal nerve with absent corneal and conjunctival reflexes on the same side. The examination also revealed tenderness over the right temporomandibular joint, muscles of mastication which are supplied by the mandibular division of the right trigeminal nerve (V3), and upper molars on the right side (supplied by V2). There was no deviation of the angle of mouth, pooling of saliva, inability to completely close the eye, or loss of taste sensation in one half of the tongue. Tests for cerebellar and vestibular function showed no deficit. Additionally, Rinne’s and Weber’s tests for hearing assessment yielded normal results. There were no other findings on clinical examination. Otoscopic and naso-endoscopic examinations were normal. Pure-tone audiometry revealed no sensory or conductive hearing loss on both sides (Figure 1). A dental opinion was obtained with oral examination revealing no significant pathology in the right molars and temporomandibular joint.

**Figure 2:**
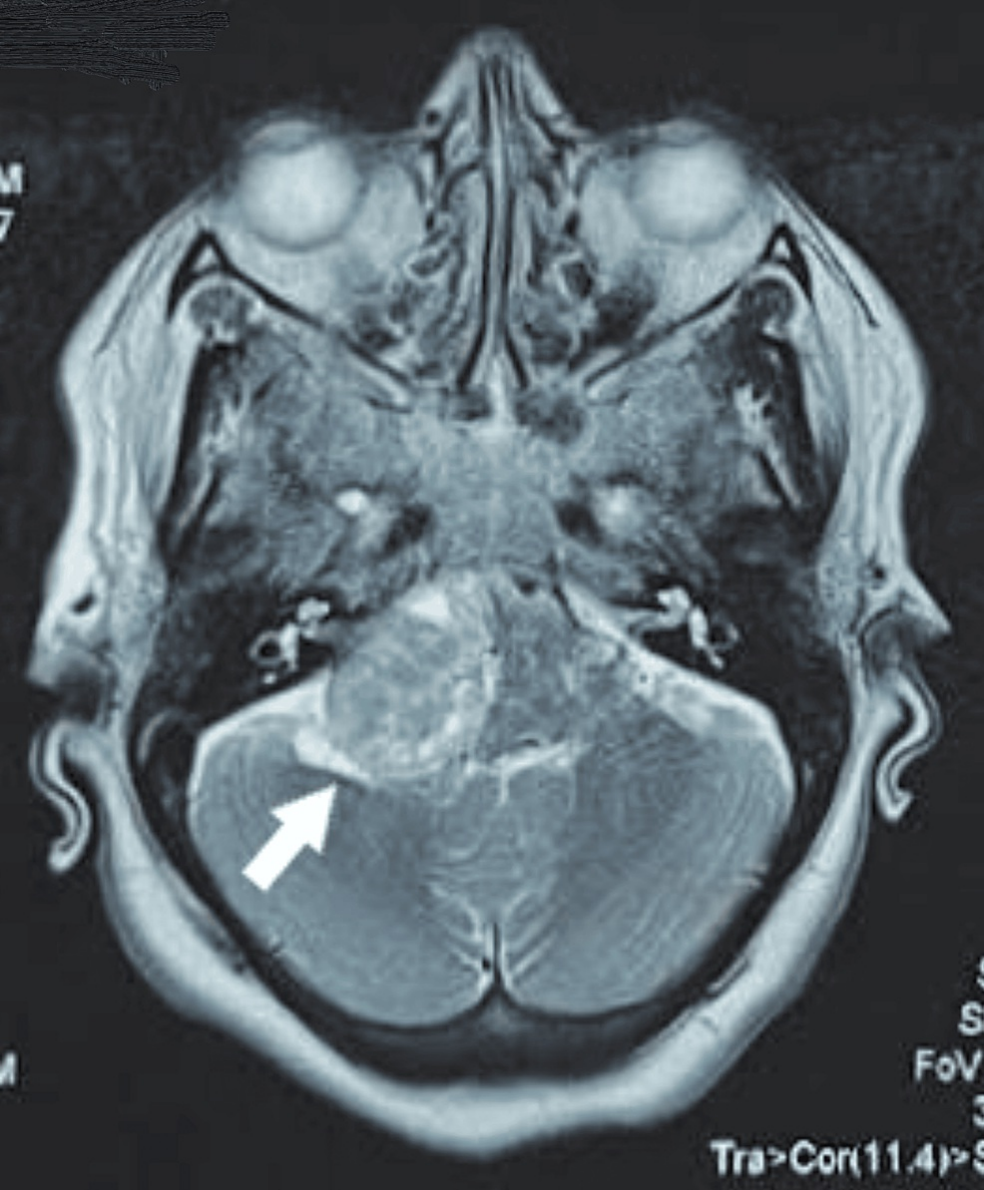
MRI Brain (transverse section) at the level of internal acoustic meatus showing typical "ice-cream cone" appearance of the tumor, compressing structures at the cerebellopontine angle .

**Figure 3:**
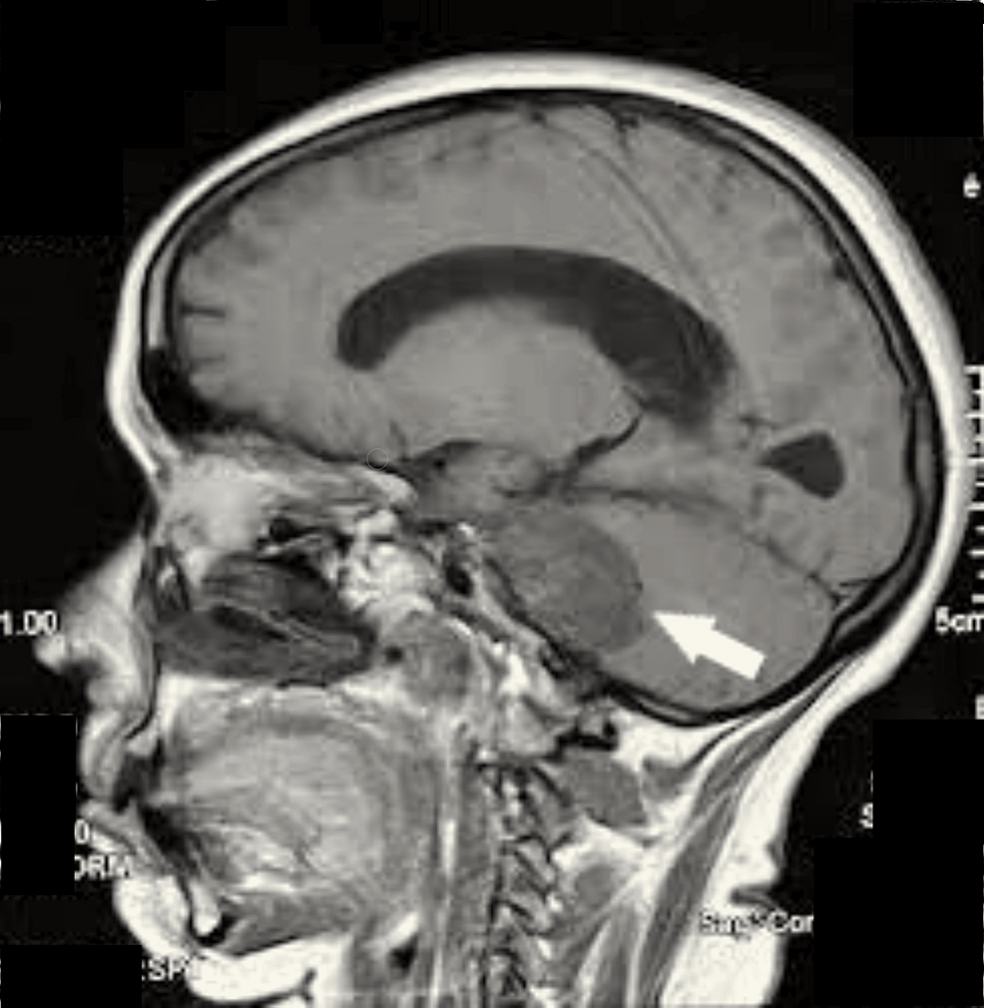
MRI Brain (sagittal section) showing the hypointense lesion compressing the cerebellum

An MRI scan of the brain was taken, which revealed a large well-defined T1 hypo intense and T2 hyper intense extra-axial mass lesion measuring 2.9 x 3.1 x 2.6 cm in the right cerebellopontine angle arising from the internal acoustic meatus growing posteromedially in the posterior cranial fossa with an ice-cream cone appearance (Figure 2) causing compression of right cerebellar hemisphere, pons and medulla (Figure 3). 

**Figure 4:**
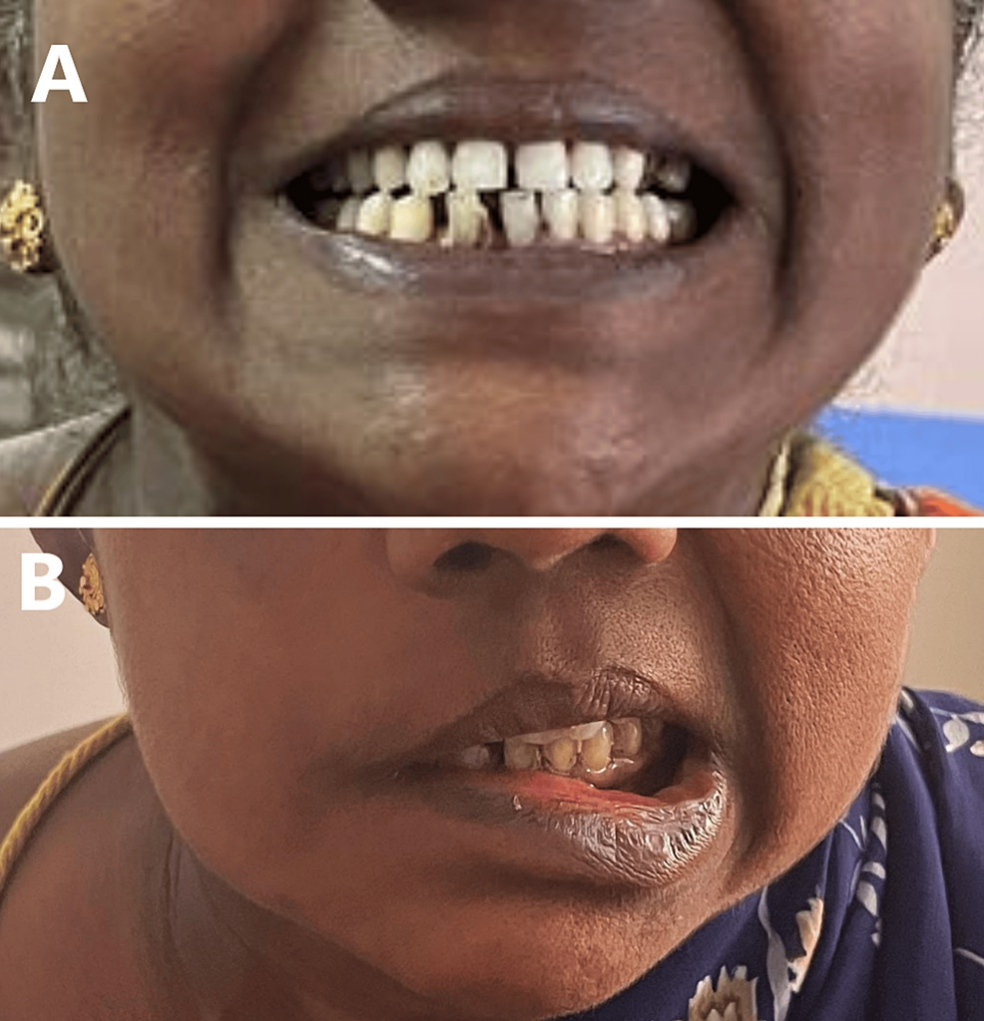
Facial nerve examination of the patient (A) Preoperative picture of the patient shows no deviation of angle of mouth; (B) Postoperative picture of the patient shows left angle of mouth deviation, suggestive of right facial nerve palsy.

**Figure 5:**
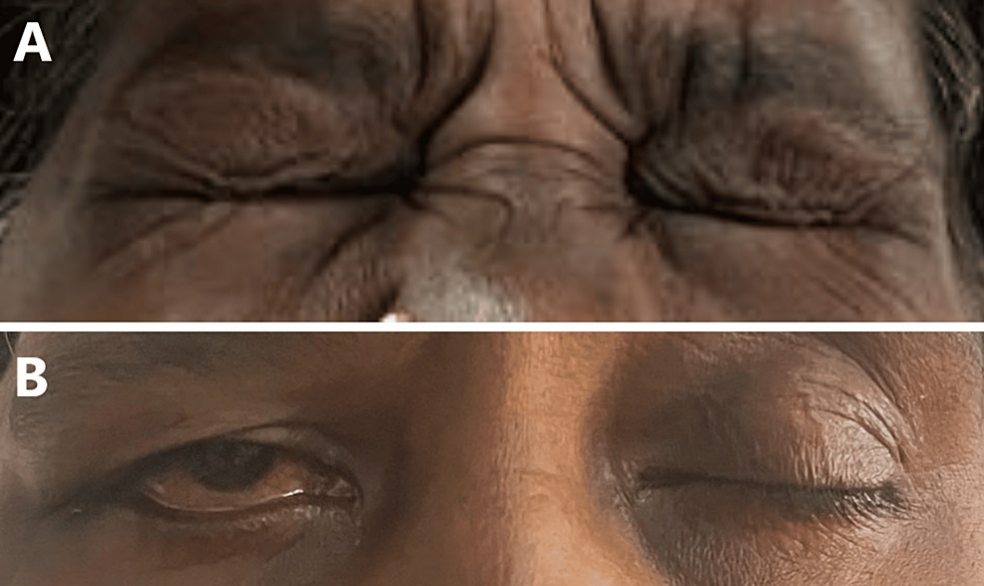
Facial nerve examination of the patient (A) Preoperative image of the patient shows ability to completely close the eyes; (B) Postoperative image of the patient shows incomplete right eye closure with uprolling of eyeball (Bell's phenomenon).

The patient was referred to the neurosurgery department and planned for a craniotomy with total excision of the tumor. The cerebellopontine angle mass was approached from a retromastoid suboccipital incision followed by a durotomy. Intraoperatively the tumor was noted to be grossly a greyish-white, firm, vascular, capsulated mass. A total excision of the tumor was done. Histological analysis of the tumor mass revealed fragments of benign neoplasm composed of sheets and fascicles with hypocellular areas and hypercellular areas showing slender spindle cells arranged in Antoni A and B pattern interspersed by thin-walled capillaries with no evidence of atypia, hence suggestive of Schwannoma. Postoperatively, the patient developed features suggestive of facial nerve palsy: left deviation of angle of the mouth (Figure 4), Bell’s phenomenon of the right eye (Figure 5), loss of right nasolabial crease, and inability to fully close the right eye (House-Brackmann grade VI). 

Along with postoperative care, physiotherapy was given to the affected side of the face, and the patient was advised to follow up regularly. She was followed up for six months, during which she had a complete recovery of her tooth pain and facial numbness with a partial recovery of the facial nerve (House-Brackmann grade III). 

## Discussion

Acoustic neuromas within the internal acoustic meatus usually cause vestibular signs and symptoms. Most of the other atypical symptoms of acoustic neuroma are caused by mass effect when it extends beyond the meatal orifice. Vestibular schwannoma has the following symptoms: hearing deficits (60-97%), tinnitus (50-66%), numbness of the face (8-10%), dizziness (20-25%), Bell’s palsy (15-20%), trigeminal disturbances like paresthesia, hyperesthesia, and neuralgia (10-15%). Patients usually pay little heed to these symptoms and it takes 0.6-5 years on average to seek medical care. Hearing impairment is not present in 8% of cases and tumor is diagnosed only based on radiological investigations [6]. In the present case, there was no hearing impairment.

To date, there are very few case reports [1,7] in the literature of acoustic neuroma mimicking facial paresthesia, whereas most of the published work [8-11] is on trigeminal neuralgia or oromandibular pain secondary to acoustic neuroma. When the tumor reaches a size of 2 cm, it extends out of the acoustic meatus and starts to compress the trigeminal nerve [12]. Acoustic neuroma-related trigeminal neuropathy is caused by direct tumor pressure on the nerve or an artery in contact (superior cerebellar artery) causing demyelination of somatosensory fibers [4]. In the present case, there was decreased sensation over the distribution of the ophthalmic nerve (V1) and maxillary nerve (V2) along with tenderness over the distribution of the mandibular nerve (V3). Nerve compression can simultaneously cause pain due to irritated/damaged fibers and numbness from hampered sensory transmission.

A patient presenting with isolated trigeminal nerve palsy should be screened for demyelinating disorders (multiple sclerosis), autoimmune conditions (systemic lupus erythematosus, Sjogren’s syndrome, sarcoidosis), metabolic conditions (diabetes mellitus) and intracranial tumors. In the current patient, all other causes were ruled out by appropriate lab and radiological investigations. MRI is the most effective tool in detecting intracranial tumors and has the advantage over CT in identifying soft tissues and nerve paths. The ability to visualize multi-layer sections contributes to better assessment of different segments of the trigeminal nerve. The increase in the incidence of acoustic neuroma cases presenting with atypical symptoms in the last two decades is attributed to the frequent use of more sensitive magnetic resonance techniques, diagnosing even small tumors [13]. MRI images of acoustic neuroma show a typical “ice cream cone sign”, which is a distinguishing feature between acoustic neuroma and other cerebellopontine angle tumors [4]. In the present case, the MRI picture had that typical appearance. The size of the tumor is measured best using the American Academy of Otolaryngology-Head and Neck Surgery (AAO-HNS) method where the axial image with the largest extra canalicular tumor diameter is selected and the maximum anteroposterior and mediolateral tumor diameters are calculated. The size of the tumor is calculated as the square root of the product of these two diameters [14].

Early diagnosis of acoustic neuroma is the most important factor in preventing its serious consequences. Management options for acoustic neuroma include observation, radiosurgery, or microsurgical excision of the tumor. Some authors advise conservative management for patients with small lesions, minimal symptoms, and those who are not able to undergo surgery. However, these patients should be under constant supervision. Tumors measuring <1 cm are managed conservatively with yearly MRI for five years, followed by a biennial MRI for four years, and one last MRI five years later, after which no further radiological follow-up is required. This is because most of the tumors grow within five years [3]. Radiosurgery has shown better outcomes in various studies with an improvement in facial nerve function and preservation of the vestibulocochlear nerve [15]. However, microsurgery was found to be superior to radiosurgery in Acoustic neuroma patients with a tumor size of >3 cm and with trigeminal nerve compression [16].

Postoperative complications are commonly observed after surgery. About 22% of patients experience at least one significant postoperative complication, of which facial paralysis is found in 14% [17]. Other postoperative complications include persistent headache, loss of hearing, cerebrospinal fluid leak, and rarely anesthesia dolorosa [18]. Regarding facial nerve recovery after surgery, the rate of good outcome was 90% at the one-year follow-up if no intraoperative facial nerve damage was observed, the tumor size was less than 30 mm, and there was no evidence of hydrocephalus [19].

## Conclusions

Acoustic neuromas, usually linked to hearing loss, can present with facial numbness and pain. This case highlights a rare presentation without hearing impairment. MRI's strength in detecting small tumors is crucial for such atypical cases. Early diagnosis is essential to avoid complications like facial nerve palsy experienced by this patient after surgery. This case adds to the growing body of evidence on atypical acoustic neuroma presentations identified through advanced imaging techniques. Clinicians should be aware of such atypical symptoms indicating a serious underlying pathology, and radiological investigations should be sought as soon as possible for early diagnosis and appropriate management.
